# Advances and Challenges in European Paediatric Palliative Care

**DOI:** 10.3390/medsci8020020

**Published:** 2020-04-17

**Authors:** Lorna K Fraser, Myra Bluebond-Langner, Julie Ling

**Affiliations:** 1 Martin House Research Centre, University of York, York YO10 5DD, UK; lorna.fraser@york.ac.uk; 2 Palliative Care for Children and Young People, Louis Dundas Centre, UCL Great Ormond Street Institute of Child Health, London WC1N 1EH, UK; bluebond@ucl.ac.uk; 3 European Association for Palliative Care, 1800 Vilvoorde, Belgium

**Keywords:** palliative care, paediatric/pediatric, child, life-limiting, decision-making

## Abstract

Advances in both public health and medical interventions have resulted in a reduction in childhood mortality worldwide over the last few decades; however, children still have life-threatening conditions that require palliative care. Children’s palliative care is a specialty that differs from palliative care for adults in many ways. This paper discusses some of the challenges, and some of the recent advances in paediatric palliative care. Developing responsive services requires good epidemiological data, as well as a clarity on services currently available and a robust definition of the group of children who would benefit from palliative care. Once a child is diagnosed with a life-limiting condition or life-limiting illness, parents face a number of complex and difficult decisions; not only about care and treatment, but also about the place of care and ultimately, place of death. The best way to address the needs of children requiring palliative care and their families is complex and requires further research and the routine collection of high-quality data. Although research in children’s palliative care has dramatically increased, there is still a dearth of evidence on key components of palliative care notably decision making, communication and pain and symptom management specifically as it relates to children. This evidence is required in order to ensure that the care that these children and their families require is delivered.

## 1. Introduction

Children’s palliative care is a specialty that encompasses the care of children with life-limiting or life-threatening conditions regardless of their diagnosis or stage of illness. Entering the fourth decade of research [1], this paper explores some of the issues which have shaped the development of paediatric palliative care, highlights some of the advances and challenges, and looks forward to what is required to keep pace with the changing landscape in medicine and scope of care and treatment for children and their families.

## 2. Key Aspects of Paediatric Palliative Care

Children’s palliative care is an evolving specialty that differs significantly from palliative care for adults in several ways [2]:

- The number of children dying is small compared to adults, with many of the conditions being extremely rare, and many of the diagnoses specific to childhood.
- The time scale of children’s illnesses differs to that of adults. In children, palliative care may be required for only a few days, months or can in some cases extend over many years. Life-limiting conditions in children can be familial; therefore, they may affect more than one child in the family.
- The focus of care is not only the child, but it also embraces the whole family. Often parents are expected to become providers of healthcare for children with very complex needs, especially those who are technology-dependent. Paediatric palliative care can offer support in a situation where the parents and siblings are especially vulnerable.
- Despite a diagnosis of a life-limiting condition, the children continue to develop physically, emotionally and cognitively. Of particular concern are the child’s communication skills and their ability to understand their condition. Provision of education and play when a child is unwell is essential and education is a legal entitlement in many countries [2].

Various international organisations and policy documents draw attention to the differences between adult and paediatric palliative care, most notably the World Health Organization (WHO). The WHO has always recognised that paediatric palliative care is different from adult palliative care and importantly states that ‘…It begins when illness is diagnosed, and continues regardless of whether a child receives treatment directed at the disease’ [3]. 

Life-limiting and life-threatening conditions are terms often used to define the population of children who would benefit from input from paediatric palliative care services (see Box 1). Life-limiting conditions are those for which there is no reasonable hope of cure and from which children or young people will die. Some of these conditions cause progressive deterioration rendering the child increasingly dependent on parents and carers [2]. Life-threatening conditions are described as those for which curative treatment may be feasible but can fail, such as cancer. If children are in long-term remission or have received successful curative treatment are no longer included [2]. Others suggest that encompassing the terms life-limiting and life-threatening results in a very heterogeneous group of diagnoses with nearly four hundred individual diagnoses classified as life-limiting or life-threatening in children [4]. However, not all serious illnesses in childhood are encompassed by the terms life-threatening or life-limiting. A new definition of palliative care put forward by the International Association of Hospice and Palliative Care [5] combines adult and paediatric palliative care; however, this development may be less useful for research, clinical practice, service development and delivery, and hamper lobbying and advocating specifically on behalf of children. It has not been endorsed by the European Association of Palliative Care (EAPC), nor by several other regional, national and international palliative care organisations.

Box 1 **Together for Short Lives/ACT Categories (TFSL 2018).**

Category 1: Life-threatening conditions for which curative treatment may be feasible but can fail, where access to palliative care services may be necessary when treatment fails, irrespective of the duration of that threat to life. On reaching long-term remission or following successful curative treatment there is no longer a need for palliative care services.
  Examples: Cancer, organ failures of heart, liver, kidney, transplant and children on long-term ventilation.
Category 2: Conditions where premature death is inevitable, these may involve long periods of intensive disease-directed treatment aimed at prolonging life and allowing participation in normal activities. Children and young people in this category may be significantly disabled but have long periods of relatively good health.
  Examples: Cystic fibrosis, Duchenne muscular dystrophy and SMA Type 1.
Category 3: Progressive conditions without curative treatment options, where treatment is exclusively palliative and may commonly extend over many years.
  Examples: Batten disease, mucopolysaccharidoses and other severe metabolic conditions.
Category 4: Irreversible but non-progressive conditions causing severe disability leading to susceptibility to health complications and likelihood of premature death. Palliative care may be required at any stage and there may be unpredictable and periodic episodes of care.
  Examples: Severe cerebral palsy, complex disabilities such as following brain or spinal cord injury.

## 3. Service Development and Accessibility to Paediatric Palliative Care in Europe

**Figure 1:**
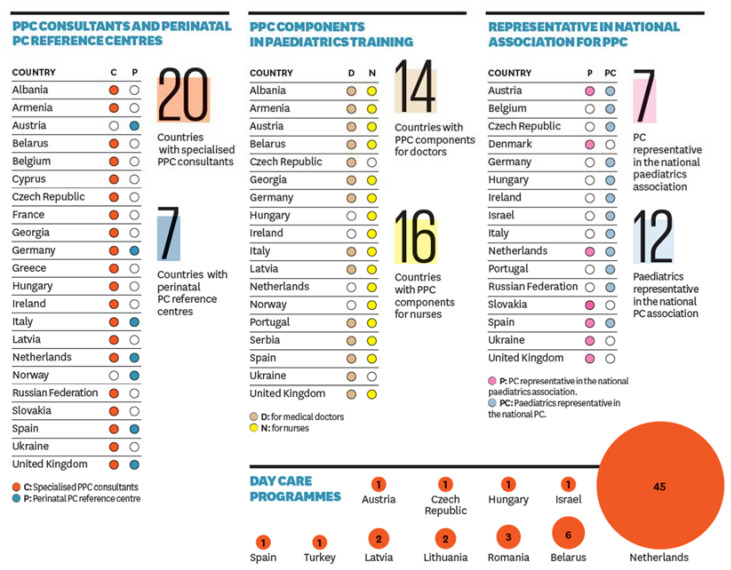
Paediatric palliative care development in Europe [6].

The recently published European Association of Palliative Care (EAPC) Atlas on Palliative Care in Europe [6] included an overview of children’s palliative care developments in Europe for the first time. The Atlas reports on specific country data in five areas: national policy, the provision of education, access to and the use of essential palliative care medicines, the provision of palliative care services and evidence of professional activity. Results shown in the Atlas illustrate the number of countries where palliative care for children is being developed with specialised paediatric palliative care consultants in twenty countries, and training in the specialty available to doctors in fourteen countries and for nurses in sixteen countries [6] (Figure 1). These developments are welcome, but there are still many countries in the WHO Europe Region where palliative care for children is not available. Improvements in technology and healthcare have resulted in many children with life-limiting conditions living longer, and it is this group that will require access to palliative care services.

Advances in both public health and medical interventions have resulted in a reduction in childhood mortality worldwide over the last few decades. Despite this, 6.6 million children (0–14 years) die every year [7]. Understanding the epidemiology of the population of babies, children and young adults who would benefit from paediatric palliative care services is important for policy, strategy, service planning and development, but it is challenging, with varying definitions and changing populations.

## 4. Changing Populations

Although deaths in childhood have been decreasing, there are still approximately 5500 infants and children who die in the United Kingdom each year [8]. Infant mortality is higher than other similarly situated countries and rising [9]. While the mortality in paediatric intensive care units (PICU) has also declined to 3.7%, one in six children who die will die in PICU [10]. These children also have longer stays in PICU before they die [11]. 

There is no consensus on the proportion of children who have died who could have benefited from palliative care; however, estimates include 50% in the UK [8] or 80% in the US [12]. There is no clear evidence to explain why 100% of children who have died would not have benefitted from palliative care, particularly given the prognostic uncertainty and the requirement for focused professional guidance urging parallel planning and studies demonstrating its utility. 

Whilst end of life care is a key component of paediatric palliative care, children often live for many years with their life-limiting or life-threatening condition. Meeting their ongoing and longer-term palliative care needs often requires increased involvement and support from specialist and non-specialist palliative care services in different ways, often in tandem with specialist disease directed services as care and treatment in those specialities change with advances in medicine and technology.

**Figure 2:**
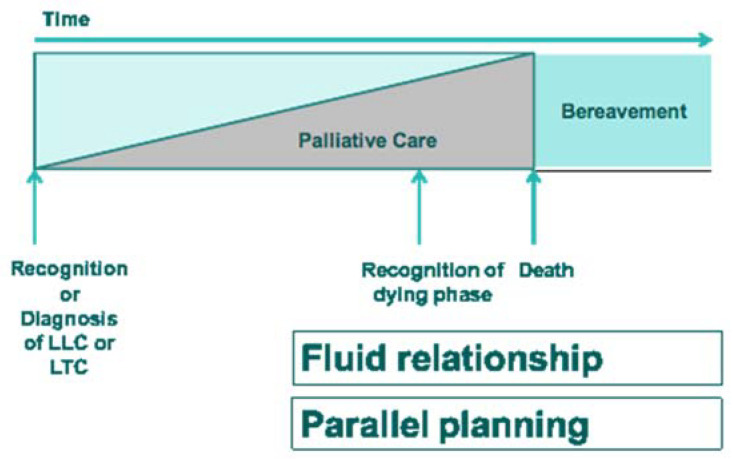
Model of Paediatric Palliative Care.

One model for addressing these issues is shown as Figure 2.

The population of children with life-limiting or life-threatening conditions is changing. For example, many of the children with neurological conditions are living longer with increasing health and care needs, including increased use of medical technologies such as long term home ventilation [13] and gastrostomy tubes [14]. This population also accounts for nearly 60% of all PICU admissions [15]. 

With advances in research, technology and treatment (e.g., the use of gene therapy, or Nusinersen for spinal muscular atrophy [16]) survival is increasing. Ironically, perhaps this increase in survival may result in a greater rather than decreased need for palliative care services. Identifying and assessing what services will be needed and when, will require more robust data than is currently available. Routine data currently collected lack detailed information on the complexity, severity and needs of the children and their conditions. Data often exclude what happens when children reach adulthood and need to transition and access adult services. Non-malignant diagnoses are common in children and young adults, and palliative care services that have historically focused on oncological care need to widen their remit to serve this population of life-limited patients [17]. Other non-palliative care services are also required in the care of these young adults. 

Place of death has been used in policy documentation as a measure of quality of palliative or end of life care in developed countries such as the United Kingdom. Both the appropriateness of using place of death, and the assumption that everyone wants to die at home has been contested in studies in both children’s and adult palliative care [18,19,20]. Further research is needed at individual/family, population and service levels if we are truly going to be able to offer choice.

In a recent national study from England and Wales, palliative care input was shown to be associated with more children dying outside of the hospital system. Those who died after being discharged from paediatric intensive care units and had input from palliative care were eight times more likely to die in the community than children who were not referred to palliative care [21]. However, there is also evidence that some children, such as those with haematological malignancies, are less likely to be referred to palliative care [22]. Recently initiated work on the development of a children’s palliative care outcome scale (CPOS) study [23] should go some way to addressing this issue. However, further higher quality, more detailed routine data collection within all health and care services is required in order to best evaluate both the need for paediatric palliative care services and the effectiveness of their delivery.

## 5. Communication and Decision Making with Children, Young People and Parents

When a child is diagnosed with a life-limiting condition or life-limiting illness, parents face a number of a complex and difficult decisions, not only about care and treatment, but also about the place of care and how this will be delivered both when the child is doing well and when the child’s condition deteriorates [24]. Support in decision-making is a key element of paediatric palliative care; not simply because of the number and complexity of decisions to be made, or even the context in which they are made, but rather because all must live with the decisions they make. 

As with decision-making in adult palliative care—in all decision-making for that matter—there are two essential elements: defining the problem [25], and identifying goals and preferences [26,27]. Proper medical decision-making requires that the patient or the patient’s family be given enough information so that they can develop an adequate understanding of the problem and the options available for responding to it. 

This passing on of information, essential to the definition of the problem, is both complex and challenging. The individual to whom the information is being passed is not simply receiving it, as something poured into an empty vessel. The clinician formulates the problem in a particular way and the family is also actively interpreting that information.

The appropriate framing of the problem, communication of information, identifying goals and preferences is difficult enough to navigate, add to it the emotional context in which decision-making takes place and challenges increase. Factor in that the individual about whom the decision is to be made is a child and the decision maker a parent, the challenges rise exponentially.

The role of parents and the nature of their participation in decision making for, and with, their children is unique. Parents’ own goals and preferences do not feature in the decision process in the same way that they would for someone deciding for themselves alone. Research has found that parents’ behaviour in caring for their seriously ill children is powerfully guided by their understanding of their role [28]. This role is defined by two aspects: nurturer/protector and advocate.

Law and ethics see the parents’ role from a different perspective. In these contexts, parents are often referred to as surrogate decision makers. In healthcare, a surrogate is usually deemed to be someone who stands in place of another to represents their views and opinions. However, in the case of children this is only possible when they are at a point of understanding their illness, so that their views can be given weight. Many seriously ill children are too young for this to happen and some ill children will, because of their condition and/or its treatment, never achieve this ability. To deal with these circumstances, the term surrogate is given a second interpretation, according to which a surrogate is someone who acts in the best interests of another person.

This principle is not merely a guide for parents in making decisions about their children, it is also a standard to which their decisions can be held and, on the basis of which, be challenged by others. It is a standard widely employed throughout North America, the UK and Europe, supported by article 3 of the Convention on the Rights of the child (CRC) [28,29,30,31]. Notably, the CRC also states that children should be able to express their views about matters concerning them, and that these views be given due weight. Professional guidance for physicians in many countries also advocates the involvement of children and young people in their healthcare decisions. However, how the CRC principles and professional guidance are put into practice can and does vary substantially within institutions, let alone countries.

Bioethics has also been an active advocate for the inclusion of children and young people in discussions and decisions about their care and treatment. The core principle from which the ethical requirement to give adults control over the treatment they receive through consent is respect for autonomy. As competence is associated with autonomy, bioethicists argue that children and young people should be given an appropriate role in decision making as their understanding and judgment develops.

Both the child rights approach and the bioethical approach are attempts to map out a role for children and young people in decisions about their care by building on principles. However, empirical studies of both children and adults show that a relational approach to autonomy and decision-making is what is practiced and preferred.

For children and young people, involvement and participation in decision making is not so much about being the final arbiter or decision maker or having the final say, but rather about having their views heard, occupying the role they prefer, and being given the information they want in the way they wish to receive it [29,30,32,33,34,35,36,37,38,39].

Given that decision making is a process not just within a person’s mind, but between two or more people, there is always potential for disagreement. Once profound disagreements occur, views can become entrenched and resolution difficult and painful.

Clinicians’ and parents’ understanding of the child’s condition do not always change in step; in part because the clinician is using information, not yet obvious or known to the parents (e.g., scans, prior experience with such children), whereas the parent is thinking in terms of the child before them, their appearance and behaviour—both of which may not have changed despite indications on the scan [40]. Hence, fine-grained, detailed descriptions of what to expect in the short term as well as long term, rather than broad labels, is essential in helping parents envision what the choice they are making truly amounts to [41]. 

Writing from the standpoint of advance care planning [42], found that it is having discussions that matter, not achieving an end. Studies such as those noted above point to the need for mixed-method studies featuring analyses of audio- or video-recorded interactions among all stake holders over the course of the trajectory as they unfold in real time as a first step in developing effective guidance and interventions for supporting parents, children and young people in decision-making [40,43].

## 6. Conclusions

This article has highlighted recent advances and challenges in the field of paediatric palliative care. As a relatively new and evolving specialty, the current situation in terms of service provision and development have been described. The challenges associated with defining the population served by paediatric palliative care have been discussed. European data suggest that there is inequity in the provision of services, and that the gaps in services need to be addressed. Service providers should consider the issues raised, such as education and training for those providing paediatric palliative care, defining their population, decision-making and communication. The need for the development of services is clear, and they ought to be considered in the context of the overall healthcare system. There needs to be a stronger emphasis on further research, and importantly on the routine collection of high-quality, standardised data. Finally, although research in children’s palliative care has dramatically increased, there is still a dearth of evidence to support many of the key components of palliative care—notably, decision making, communication and pain and symptom management. This need for evidence is one of the biggest challenges for paediatric palliative care, where there is a need to ensure that children with life-limiting conditions and their families receive the best care possible.
